# The effects of human volatiles produced by skin microbiota on *Forcipomyia* (*Lasiohelea*) *taiwana* host preference

**DOI:** 10.1002/ps.70089

**Published:** 2025-07-30

**Authors:** Tengfei Lu, Yuling Zhang, Dan Gou, Haocong Chen, Xiaohui Hou

**Affiliations:** ^1^ School of Preclinical Medicine Zunyi Medical University Zunyi China

**Keywords:** biting midges, human skin bacteria, human skin volatile, host preference

## Abstract

**BACKGROUND:**

Midges are widely distributed globally. They can transmit numerous serious diseases when biting hosts. It is crucial for developing more effective midge monitoring and control methods to better understand how host volatiles attract and repel these blood‐sucking insects.

**RESULTS:**

In this work, volatile substances from human skin were detected by means of gas chromatography–mass spectrometry (GC–MS). A total of 25 compounds in relatively high contents were identified from the gauzes adsorbing volatiles of eight volunteers, including ketones, aldehydes, alcohols and acids. Nonanal, 6‐methyl‐5‐hepten‐2‐one and decyl aldehyde were all present in the skin volatiles of the volunteers, at relatively stable and high levels. Our results showed that 0.001% geranylacetone exhibited the highest attraction rate for midges, at ≤72.1%, and that 10% 3‐methyl‐1‐butanol has the highest repellent rate towards midges, reaching 70.7% by behavioral experiments. Thirty‐five types of bacteria from the skin surface of the volunteers were identified. It was discovered that 3‐methyl‐1‐butanol was mainly produced by *Staphylococcus hominis* subsp. *novobiosepticus* (R2A01–07) with a high rate of 81.898%.

**CONCLUSION:**

In summary, volatile substances can attract or repel midges in the appropriate concentration ranges. Differences in human volatile compounds are mainly regulated by the skin microbiota, which indirectly changes the olfactory behavior of midges by regulating human odor. This work is very helpful for understanding the specific mechanisms underlying the host preference of midges. © 2025 The Author(s). *Pest Management Science* published by John Wiley & Sons Ltd on behalf of Society of Chemical Industry.

## Graphical abstract

Human skin volatiles influence midge behavior, with certain compounds acting as strong attractants or repellents. These odor differences are largely regulated by skin microbiota, offering insight into host preference mechanisms and potential for improved vector control.

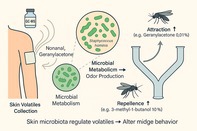

## 1 INTRODUCTION

*Forcipomyia (Lasiohelea) taiwana* (Diptera: Ceratopogonidae) are tiny blood‐sucking insects that inhabit Yunnan, Guizhou and other places in southern China. Female *F. (L.) taiwana* feed primarily on blood by biting domestic and wild ruminants, causing intense itching and swelling.^1^, ^2^ People bitten by these insects may experience varying degrees of skin inflammation, extreme itching or even severe allergic reactions depending on their physiological condition.^3^ Additionally, blood‐sucking midges have been confirmed to transmit various other bacteria, viruses, filariasis, and protozoan diseases.^5^, ^6^, ^7^ For example, Japanese encephalitis virus was obtained from biting midges.^4^ The transmission and increasing incidence of diseases caused by these pathogenic organisms also have had significant impacts on the livestock industry and people's quality‐of‐life.^8^, ^9^ Therefore, effective surveillance and control of vector blood‐sucking insects is essential for the control of vector‐borne diseases.^10^, ^11^

Vertebrate host volatiles traps were successfully utilized to monitor and control other blood‐sucking insects.^12^, ^13^ Similar techniques have been applied to control midges, achieving varying degrees of success.^14^ Surveillance programs tailored for blood‐sucking midges are essential in predicting the spread of these animal‐borne diseases. Gaining a deeper understanding of how host volatiles attract and potentially repel these insects is imperative for developing more effective monitoring and control methods for the vector insects.^9^

The blood‐sucking preference of insects has been found to primarily correlate with the type and concentration of host odor substances.^15^, ^16^, ^17^ Blood‐sucking insects detect various odor molecules in the environment with their powerful olfactory system to realize a range of behaviors such as foraging, mate seeking and oviposition site selection.^18^ It has been discovered that bovine‐derived odors could modulate the behavior of *Culicoides*, with the potential to elicit biting behavior.^9^ Likewise, mosquitoes avoid biting individuals with high levels of the volatile compound decyl aldehyde on their skin, preferring those with moderate decyl aldehyde levels.^19^ *Aedes aegypti* exhibited a stimulating response to 3‐methyl‐1‐butanol, 6‐methyl‐5‐hepten‐2‐one, geranylacetone and nonanal.^20^ Electroantennogram (EAG) studies have explored the preference for odor selection of bed bugs that feed on human blood. The results showed that bed bugs respond electrophysiologically to hexanal, (E)‐2‐octanal, octanal, nonanal, decanal, heptanal, (*R,S*)‐1‐octen‐3‐ol, 2‐decanone, dodecane and nonanoic acid from human volatile extracts.^21^ That research laid an experimental groundwork for the development of bed bug attractants.

Approximately 600 volatile organic compounds were found in human skin, but there were only four main components among them: 6‐methyl‐5‐hepten‐2‐one, decanal, nonanal and geranylacetone.^22^, ^23^ It was found that human odor can affect the host‐seeking behavior of blood‐sucking insects.^19^ Human odor is produced by resident bacteria on the skin, which convert metabolites secreted in sweat into odorants, giving the human body a unique scent.^24^ Skin microbiota is considered as the main factor contributing to human skin odor, and the significant differences in skin microbiota among individuals are the main cause of individual skin odor differences.^25^, ^26^ Olfactory sense is the primary perception method for mosquitoes to identify and locate their hosts.^18^ Many studies have demonstrated the important role of microorganisms in host identification and localization of blood‐sucking insects.^27^, ^28^, ^29^, ^30^ The main reason mosquitoes exhibit a preference for their hosts lies in the variations in the types and quantities of odor substances emitted by the hosts.^28^, ^31^ Therefore, research on host odor substances is beneficial not only for developing new attractants and repellents for midges, but also specifically for analyzing the host preference of *F. (L.) taiwana* midges in field monitoring.

Researchers have conducted a lot of work on the host preferences of mosquitoes, and corresponding knowledge about the blood‐sucking habits and feeding patterns of the midges is expanding. However, the host preferences of *F. (L.) taiwana* have not yet been described in detail. In the present study, it was found that 0.001% geranylacetone has the highest attraction rate to midges, and that 10% 3‐methyl‐1‐butanol has the highest repellent rate towards midges. Eighteen types of bacteria from the skin surface of the eight volunteers were identified. 3‐methyl‐1‐butanol was mainly produced by *Staphylococcus hominis* subsp. *Novobiosepticus*. Volatile substances can attract or repel midges in the appropriate concentration range. The differences in human volatile compounds, mainly regulated by the skin microbiota, indirectly change the olfactory behavior of midges by regulating human odor. The present results help build the foundations for future research on the blood‐sucking preference mechanism of the *F. (L.) taiwana*.

## 2 MATERIALS AND METHODS

### 2.1 Insects

The adult *F. (L.) taiwana* used in this study were collected in April–August 2022 in farmers in Guiyang City (26° 25′  N, 106° 40′ E) and Zunyi City (27°42′ N, 106°55′ E), Guizhou Province, using UV lamp traps. The collected midges were brought back to the laboratory immediately where they were fed on 5% honey solution for maintenance. They were kept at room temperature (25 ± 2 °C) and with a relative humidity of 75 ± 2% under a 12 h:12 h, light:dark photperiod.

### 2.2 Volunteer induced experiment

Eight volunteers with varying degrees of bite by midges from 285 survey respondents were selected in their daily lives. The volunteers were all males aged 20–25 without underlying diseases. Field lure experiment were conducted at the edge of rice fields in Xiaojiawan, Zunyi City by the volunteers, who were prohibited within 48 h of the test from bathing, spraying perfume, and other behaviors that affect body odor. Participants were maintained at a minimum separation distance of 1.5 m to prevent cross‐interference. The number of attracted female midges was counted every 20 min, and the lure experiment were repeated five independent times. The results were expressed as mean ± SD and analyzed by one‐way ANOVA coupled with Tukey's honestly significant difference (HSD) method. All procedures were approved by the Animal Ethics Committee of Zunyi Medical University (ZMU21‐2303‐016).

### 2.3 Volatile substance collection from human skin and gas chromatography–mass spectrometry (GC–MS)

The eight volunteers were wrapped all over their bodies in clean gauze without any organic volatile compounds. They were required to live a normal life yet without bathing, spraying perfume and other behaviors that affect body odor. Volatile compounds were obtained with head‐space volatile trapping instrument from 5 g sheared gauze. The head‐space extracts were carried out by means of GC–MS on HP6890 linked to 5975C (Agilent Technologies, Santa Clara, CA, USA). The system was equipped with a capillary column, HP‐5MS (60 m × 0.25 mm, film thickness 0.25 μm) (Agilent Technologies). The samples were injected at a constant flow rate of 1.0 mL min^−1^ in nonsplit mode. After injection, the column temperature was set at 42 °C for 2 min. Then it was increased up to 183 °C at 3.5 °C min^−1^. Subsequently, the temperature was raised to 308 °C at 10 °C min^−1^ and held for 2 min. The temperatures of the interface, ion source and quadrupole were 280, 230 and 150 °C, respectively. Each compound was detected and scanned using 70 eV electron energy in the range of 29–500 amu with a solvent delay of 3 min. Volatile components were determined and identified by comparing with standard mass spectra in the NIST and Wiley database. The relative mass fractions of each component were calculated by the area normalization method.

### 2.4 Chemicals

All volatile chemical compounds used in the manuscript were purchased from different companies. Decyl aldehyde (CAS: 112‐31‐2) (GC, 97%), Nonanal (CAS: 124‐19‐6) (GC, 95%), 3‐Methyl‐1‐butanol (CAS: 123‐51‐3) (GC, 99%), Heptaldehyde (CAS: 111‐71‐7) (GC, 95%), and 6‐Methyl‐5‐hepten‐2‐one (CAS: 110‐93‐0) (GC, 98%) were produced by TCI Development Co., Ltd (Shanghai, China). Geranylacetone (CAS: 3796‐70‐1) (GC, 98%) was produced by Bide Pharmatech Ltd (Shanghai, China). Ethanol (CAS: 64‐17‐5) (AR, 99.7%) was obtained from Tianjin Fuyu Fine Chemical Co., Ltd (China).

### 2.5 Electroantennography (EAG)

Healthy and active female adult *F. (L.) taiwana* midges were selected for the EAG test. An antenna was carefully excised from the base to expose antennae sensors with the aid of stereomicroscope (Nikon, Japan). The treated antenna was immediately set between reference glass electrodes and recording glass electrodes containing physiological saline. Fifteen microliters of reagent were dropped onto a 4 × 0.5 cm filter paper strip and the strip was placed into a 1‐mL pipette tip. A continuous flow of filtered air at 600 mL min^−1^ was delivered. The exposed antenna was stimulated every 1 min for 0.5 s each time. Each treated antenna was used to detect all concentrations of a single commercial compound. Volatile samples were measured in the order of blank group, low concentration group, high concentration group and blank group. Each compound was repeated 10 times with 10 antennas from 10 different adult midges. The volatile compounds to be tested were randomly selected with anhydrous ethanol as the blank control. Antennal activity of each compound was detected when getting a stable baseline. The EAG relative value was calculated as follows: *V* = 2*R*/(*C*_1_ + *C*_2_), where *R* is the average EAG value of the tested chemical compound, C_1_ denotes the EAG value of the anhydrous ethanol before testing and C_2_ represents the EAG value of the anhydrous ethanol after testing the compound. The detected values from different groups were analyzed by one‐way ANOVA coupled with Tukey's HSD method.

### 2.6 Behavior assays of *F. (L.) taiwana* to the volatile substances

The adult *F. (L.) taiwana* were acclimated to the testing room at 26 ± 2 °C and RH 70 ± 5% in natural light for 1 h before behavior assays. The volatile compounds were dissolved into eight different concentrations in anhydrous ethanol. Each compound was determined with five replicates in natural light. The Y‐shaped olfactometer was used to assess the behavioral response of individual midges to the compounds. The Y‐shaped olfactometer was equipped with an air pump, a gas washing cylinder, a drying tower containing activated carbon, a sample bottle, a flow meter, a silicone tube, Y‐shaped tube and insect collector. The gas flow rate was 0.4 L min^−1^ during the measurement process. Same filter papers (2.5 × 5 cm) including 100 μL test compounds or an equal amount of anhydrous ethanol as control were placed into the left and right chambers, respectively. Then 20 female adult midges were prepared in the insect collector of the Y‐shaped olfactometer. Their first choice was recorded within 5 min, or otherwise recorded as no response. The filter paper was replaced every time an experiment was conducted. The positions of the two arms of the Y‐shaped tube were swapped after two repeated measurements to eliminate the influence of the geometric position on the selection behavior. The Y‐shaped tube was thoroughly cleaned with deionized water and soap solution. The statistical results of behavioral experiments refer to the method proposed by Isberg E *et al*.^32^ The selection rate is calculated as the ratio of the number of the midges that entered either the treatment or control arm in the Y‐shaped tube test to the total number of midges that have made choices.

### 2.7 Submicroscopic observation of antennal sensory in *F. (L.) taiwana*

The antennae of adult *F. (L.) taiwana* were processed into specimens for observation by scanning electron microscope (SEM). The samples were fixed with 4% glutaraldehyde after washing with deionized water for three times. They were immersed in concentration gradient of ethanol (35%, 50%, 75%, 80%, 85%, 90% and 100%) for 15 min, respectively. The treated samples were dried by critical point drying with EM CPD300 (Leica Microsystems GmbH, Wetzlar, Germany) and were gold coated using an EM SCD050 sputter coater (Leica Microsystems GmbH). Finally, ultramicroscopic observation and photography were performed using SU8000 SEM (Hitachi High‐Technologies Corporation, Tokyo, Japan).

### 2.8 Cultivation of bacteria on the surface of human skin

Hand, forehead, elbow socket and popliteal socket skin, which are frequently bitten by female midges, were swabbed with sterile swabs over a range of 5 cm^2^. The swabs were put into tubes containing sterile water. A gradient release of 10^−2^–10^−5^ was performed on the bacterial solutions after shaking culture, and resultant solutions were cultured with Luria–Bertani and R2A solid media at 37 °C for 48 h. The purified bacterial strains after six rounds of purification underwent preliminary morphological identification. The identified bacteria were selected for Gram staining and other subsequent experiments.

### 2.9 Bacterial 16S rRNA gene amplification

Genomic DNA of the cultured bacteria were extracted with a Bacterial Genomic DNA Extraction Kit (DP302; Tiangen, Beijing, China). The 16S rRNA gene was amplified using the 27F/1492R primers. PCR products with a single band were identified for sequencing (Shanghai Sangon Biotech Co., Ltd, China).

### 2.10 Phylogenetic analysis of 16S rRNA sequence

ezbiocloud, seqman, editseq, and mega 7.0 were utilized to align, edit and analyze the 16S rRNA gene sequences of the obtained bacterial populations. A phylogenetic tree was calculated using Mega 7.0 software with *Bacteroides thetaiotaomicron* strain VPI‐5482 as the outgroup. It was constructed by the neighbour‐joining method (NJ) with the confidence values of each node based on 1000 bootstrap tests.

### 2.11 Detection of volatile substances from bacteria derived from human skin

A 1 g sample of the cultured colony was scraped from the solid culture medium. The volatile substances from bacteria were collected in a solid‐phase microextraction sampling bottle. Then, the samples were detected by GC–MS as described above. A further 1 g of uncontaminated culture medium was scraped as a blank control.

### 2.12 Statistical analysis

All quantitative experimental data were repeated at least three times and expressed as mean ± SD. prism 8.01(GraphPad Inc., San Diego, CA, USA) and Spss 29.0 (IBM, Armonk, NY, USA) software were used for plotting and statistical analysis. The data from different groups were analyzed by one‐way ANOVA coupled with Tukey's HSD method. The behavioral experimental data was analyzed using the chi‐square test.

## 3 RESULTS

### 3.1 Temptation assay

**Figure 1:**
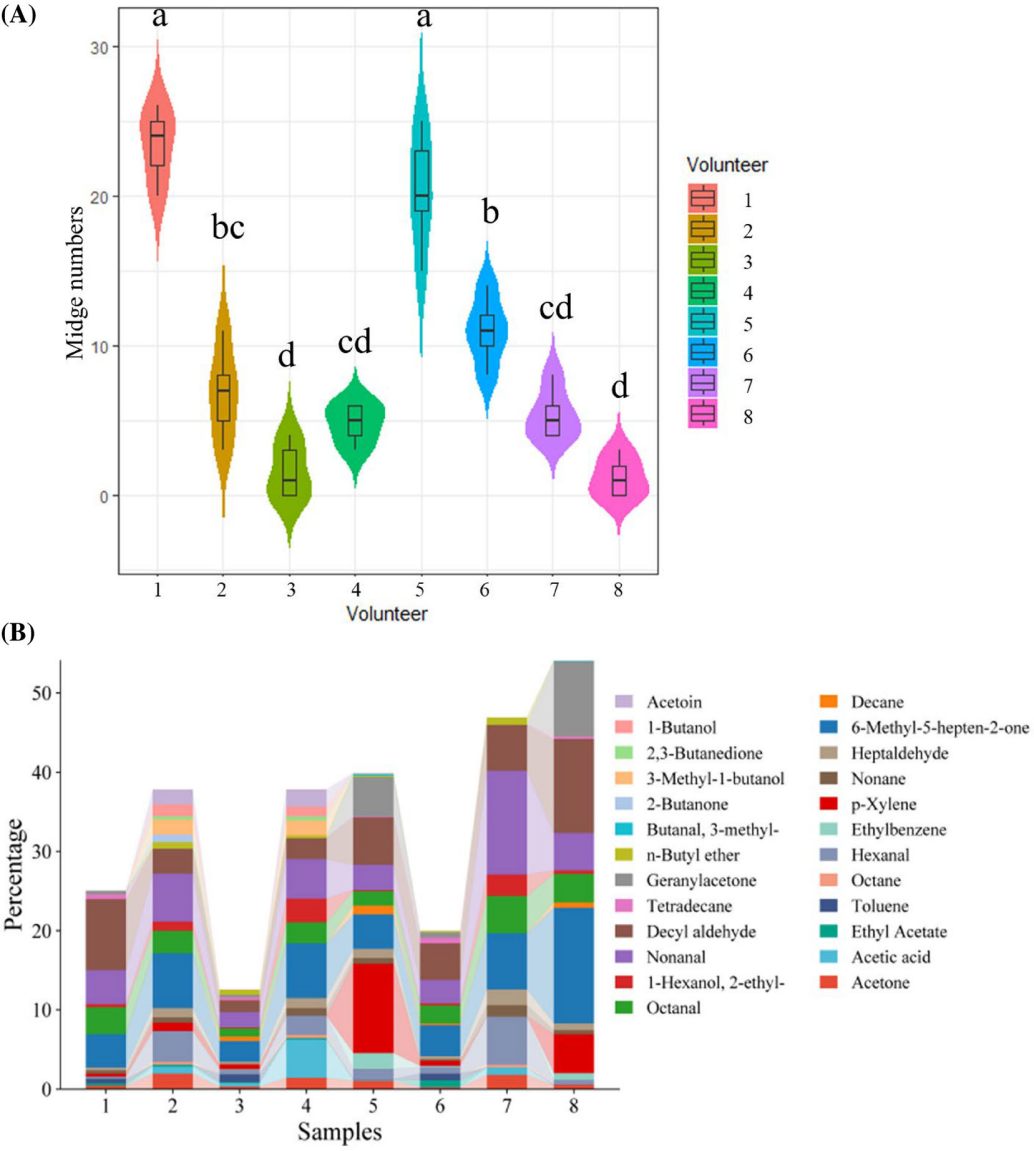
Detection of volatiles adsorbed on sterile gauze from eight volunteers attracting midges. (A) Number of *F. (L.) taiwana* attracted by eight volunteers within 20 min. The lure experiment was repeated five independent times. Results are expressed as mean ± SD. Data were analyzed by one‐way ANOVA coupled with Tukey's HSD method. Different letters in the figure represent statistical differences (*P < 0.05) between the two volunteers. The same letter or one of the two letters the same indicates no statistical difference between the volunteers (*P* > 0.05). (B) Volatiles adsorbed in the sterile gauze from eight volunteers. Volatile components were determined and identified by comparing with standard mass spectra in the NIST and Wiley database. The relative mass fractions of each component were calculated by the area normalization method. The horizontal axis represents samples from volunteers. The vertical axis represents the percentage of the volatiles in each sample.

The numbers of adult *F. (L.) taiwana* midges lured by volunteers 1 to 8 within 20 min were 23.4 ± 2.4, 6.8 ± 3.03, 1.6 ± 1.8, 4.8 ± 1.3, 20.4 ± 3.8, 11 ± 2.24, 5.4 ± 1.67 and 1.2 ± 1.3, respectively. Volunteers 1 and 5 attracted the highest number of the midges, whereas volunteers 3 and 8 attracted the lowest number of the midges. There was a statistically significant difference (*P* < 0.05) in the number of lured midges by volunteers 1 and 5, compared with that by volunteers 2, 3, 4, 6, 7 and 8 [Fig. 1(A)]. Different volunteers exhibit varying levels of attraction towards the midges, indicating that adult *F. (L.) taiwana* have different preferences for different hosts.

### 3.2 Detection of volatile substances on the surface of human skin

The gauzes of the eight volunteers [same as in Fig. 1(A)] were detected by means of GC–MS. A total of 25 compounds with relatively high contents were identified, including ketones, aldehydes, alcohols and acids. The relative content of volatile compounds in the volunteer 8 was the highest, at ≤54.06%, among which 6‐methyl‐5‐hepten‐2‐one had the highest relative content, at ≤14.62%. By contrast, the relative content of volatile compounds in volunteer 3 was the minimum, at just 12.57%, with a relative content of 6‐methyl‐5‐hepten‐2‐one of ≤2.59% [Fig. 1(B)]. The types of volatile compounds on the skin of different volunteers were similar, but the relative content of each compound is different. Among them, nonanal, 6‐methyl‐5‐hepten‐2‐one and decyl aldehyde were present in all samples from the eight volunteers, and they were relatively stable and had relatively high levels.

### 3.3 EAG response of midges to human volatiles

**Figure 2:**
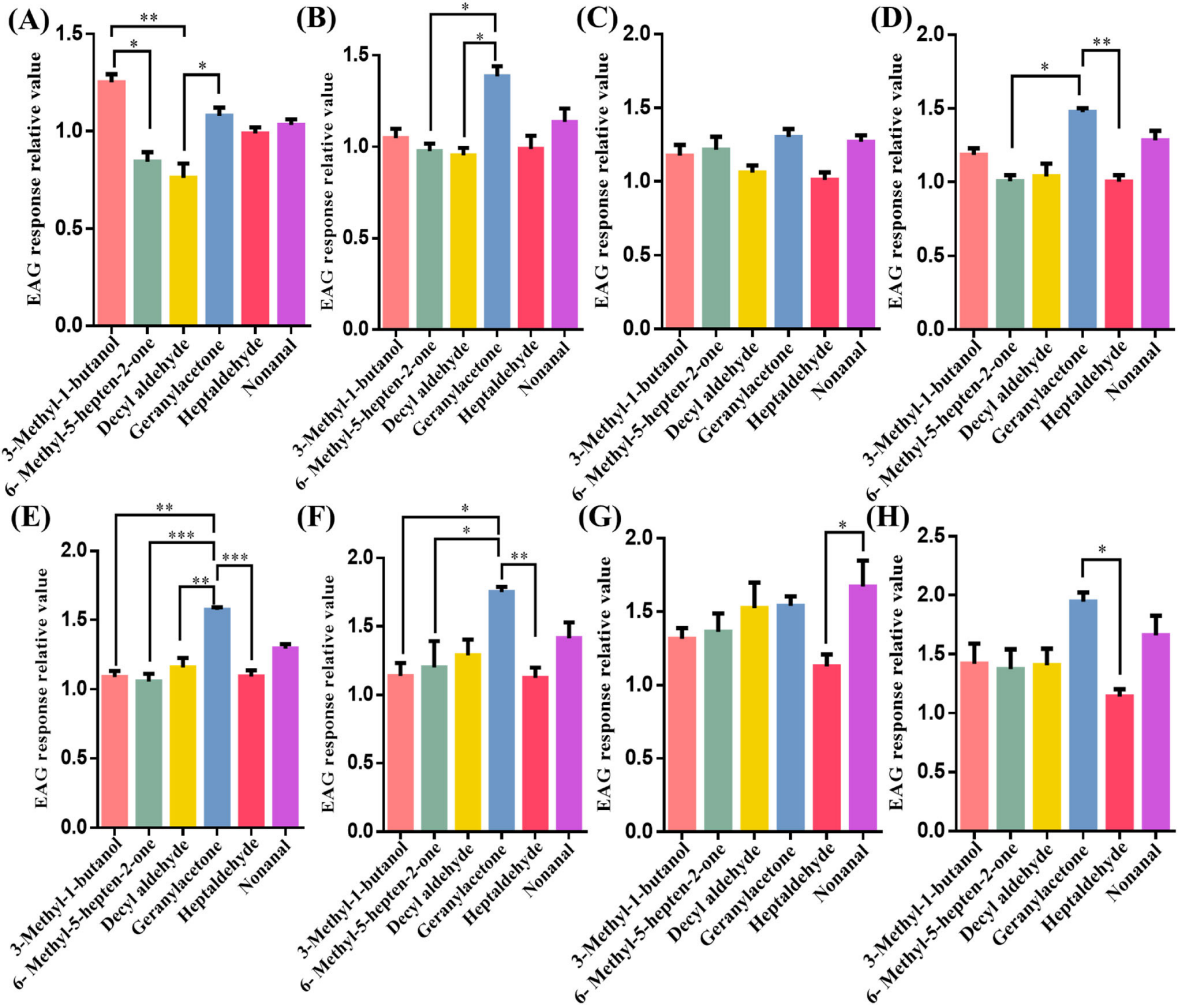
EAG values of *F. (L.) taiwana* to six different volatiles 3‐methyl‐1‐butanol, geranylacetone, decyl aldehyde, nonanal, 6‐methyl‐5‐hepten‐2‐one and heptaldehyde with different concentrations: 0.000001% (A), 0.00001% (B), 0.0001% (C),0.001% (D), 0.01% (E), 0.1% (F), 1% (G) and 10% (H). All quantitative experimental data were repeated at least three times and expressed as mean ± SD. prism 8.01 and Spss 29.0 software were used for plotting and statistical analysis. Data from different groups were analyzed by one‐way ANOVA coupled with Tukey's HSD method, with significances: **, *P* < 0.01; *, *P* < 0.05.

3‐methyl‐1‐butanol, geranylacetone, decyl aldehyde, nonanal, 6‐methyl‐5‐hepten‐2‐one and heptaldehyde were selected to perform electrophysiological stimulation on the adult *F. (L.) taiwana* based on the results above and some previous studies^20^, ^23^ of the volatile compounds of blood sucking insects. The EAG response of *F. (L.) taiwana* antenna to 3‐methyl‐1‐butanol was strongest, whereas the response to decyl aldehyde was smaller when the concentration of volatile compounds was 0.000001% [Fig. 2(A)]. The EAG values of the antennal potential response of midge to geranylacetone were higher, whereas the EAG values of the antennal response to 6‐methyl‐5‐hepten‐2‐one and decyl aldehyde were smaller [Fig. 2(B)], when the concentration of the tested volatile compounds was 0.00001%. There was no significant difference in the EAG response of *F. (L.) taiwana* antenna to different volatile compounds at 0.0001% concentration [Fig. 2(C)]. The EAG value of midge antenna treated with geranylacetone was much larger, whereas the EAG value of midge antenna treated with heptaldehyde was relatively smaller when the volatiles concentration was 0.001% [Fig. 2(D)]. The response of the *F. (L.) taiwana* antenna to geranylacetone was significantly strong, whereas the response to heptaldehyde was relatively poor when the volatiles concentrations were 0.01% [Fig. 2(E)] and 0.1% [Fig. 2(F)]. When the concentration of volatile compounds was 1% [Fig. 2(G)], the relative value of the antennal potential response of midges to nonanal was larger, whereas the relative value of the antennal potential response to heptaldehyde was smaller. The EAG value of midge antenna treated with geranylacetone was larger, whereas that of midge antenna treated with heptaldehyde was relatively smaller at 10% concentration [Fig. 2(H)].

These results showed that the midge antenna have the strongest response to 6‐methyl‐5‐hepten‐2‐one, geranylacetone, 3‐methyl‐1‐butanol and heptaldehyde at a concentration of 10%, and to decyl aldehyde and nonanal at a concentration of 1%. Overall, the stimulation response of midges showed an upward trend with the increase of volatile matter concentration, but the EAG results of 3‐methyl‐1‐butanol and heptaldehyde did not show a significant upward trend in the stimulation response. The EAG value of midges to 3‐methyl‐1‐butanol at a concentration of 0.000001% was larger, indicating that the volatile is suitable as a long‐distance stimulant in the wild. The EAG value of midges to 10% geranylacetone was much higher, indicating that it is suitable as a stimulant at close range.

### 3.4 Behavior assays of *F. (L.) taiwana* to the volatile substances

**Figure 3:**
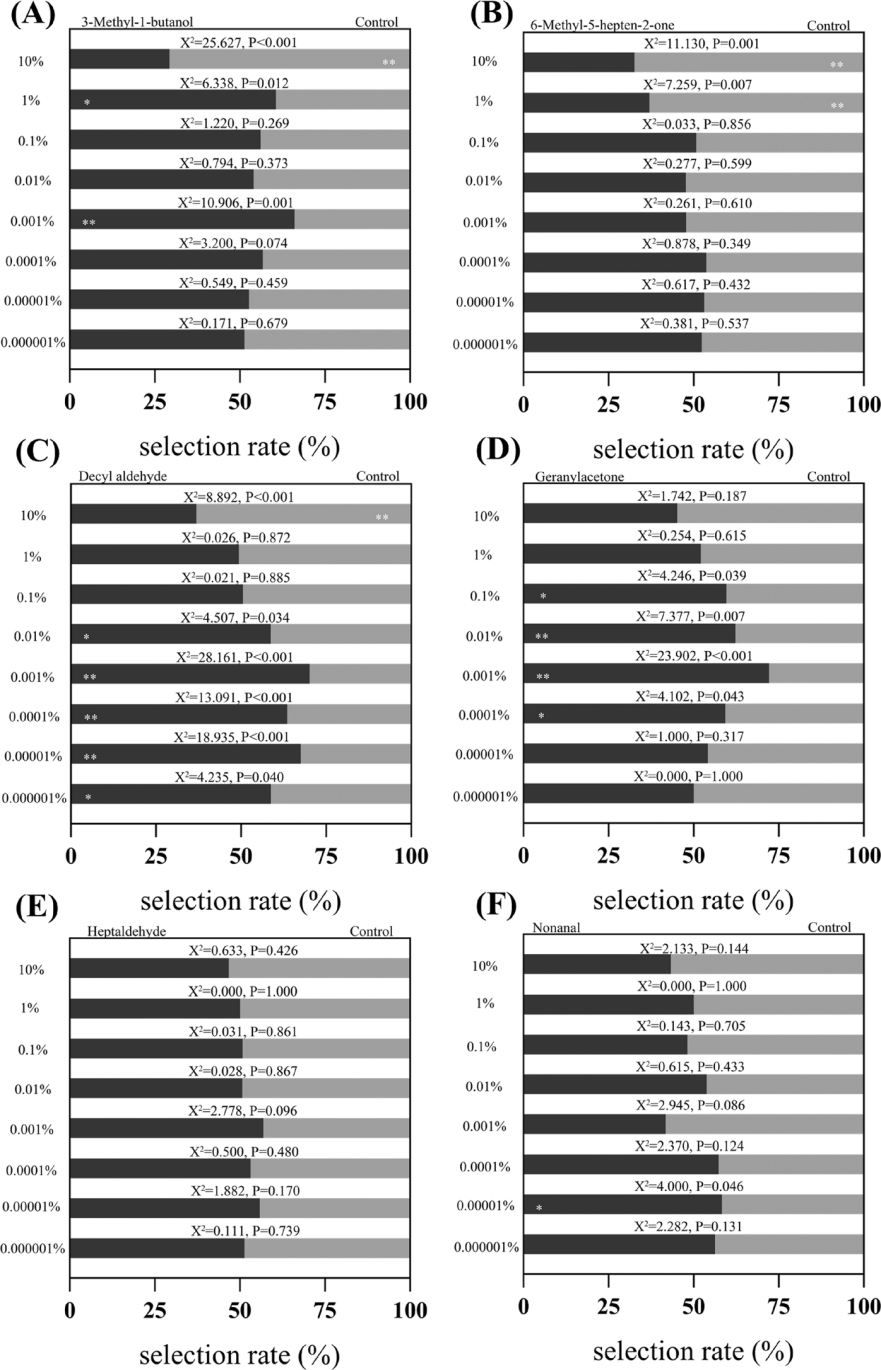
Selection of *F. (L.) taiwana* to eight different concentrations of the commercialized reagents. Behavioral response of the midges to (A) 3‐methyl‐1‐butanol, (B) 6‐methyl‐5‐hepten‐2‐one, (C) decyl aldehyde, (D) geranylacetone, (E) heptaldehyde and (F) nonanal. Each compound was determined with five replicates and expressed as mean ± SD. Behavioral experimental data were analyzed using chi‐square test between the experimental group and the control group, with significances: **, *P* < 0.01; *, *P* < 0.05.

The behavioral response of individual midges to the compounds was assessed by Y‐shaped olfactometer. 1% and 0.001% 3‐methyl‐1‐butanol were more attractive than the control group to the midges, with attraction rates of 60.6% and 66.0%, respectively. Although the control group had a more attractive effect on midges than 10% 3‐methyl‐1‐butanol [Fig. 3(A)]. The attraction of 1% and 10% 6‐methyl‐5‐hepten‐2‐one to the midges was significantly lower than that of the control reagent. Compared with the control group, different concentrations of 6‐methyl‐5‐hepten‐2‐one showed no significant attractant effect on *F. (L.) taiwana* [Fig. 3(B)]. The midges showed a preference for decanal when the concentration of decyl aldehyde was between 0.01% and 0.000001%. The attraction rates of 0.001%, 0.0001%, and 0.00001% decyl aldehyde to the midges were 70.1%, 63.6% and 67.5%, respectively [Fig. 3(C)]. When the concentration of geranylacetone was between 0.1% and 0.0001%, the midges showed a strong preference (59.3–72.1%) for geranylacetone [Fig. 3(D)]. There was no significant difference in the attraction rate in heptaldehyde and control group [Fig. 3(E)]. 0.00001% nonanal was more attractive to the midges compared with the control reagent. Other concentrations of nonanal showed no attractant effect on the midges [Fig. 3(F)]. In summary, 0.001% 3‐methyl‐1‐butanol, 0.001%, 0.0001% and 0.00001% decanal, 0.01% and 0.001% geranylacetone, and 0.00001% nonanal had strong attraction to the midge. High or low concentrations may have low attraction or even avoidance effects. Among them, 0.001% geranylacetone had the highest attraction rate to midges, at ≤72.1% (Fig. 3).

### 3.5 Identification of human skin bacteria

**Figure 4:**
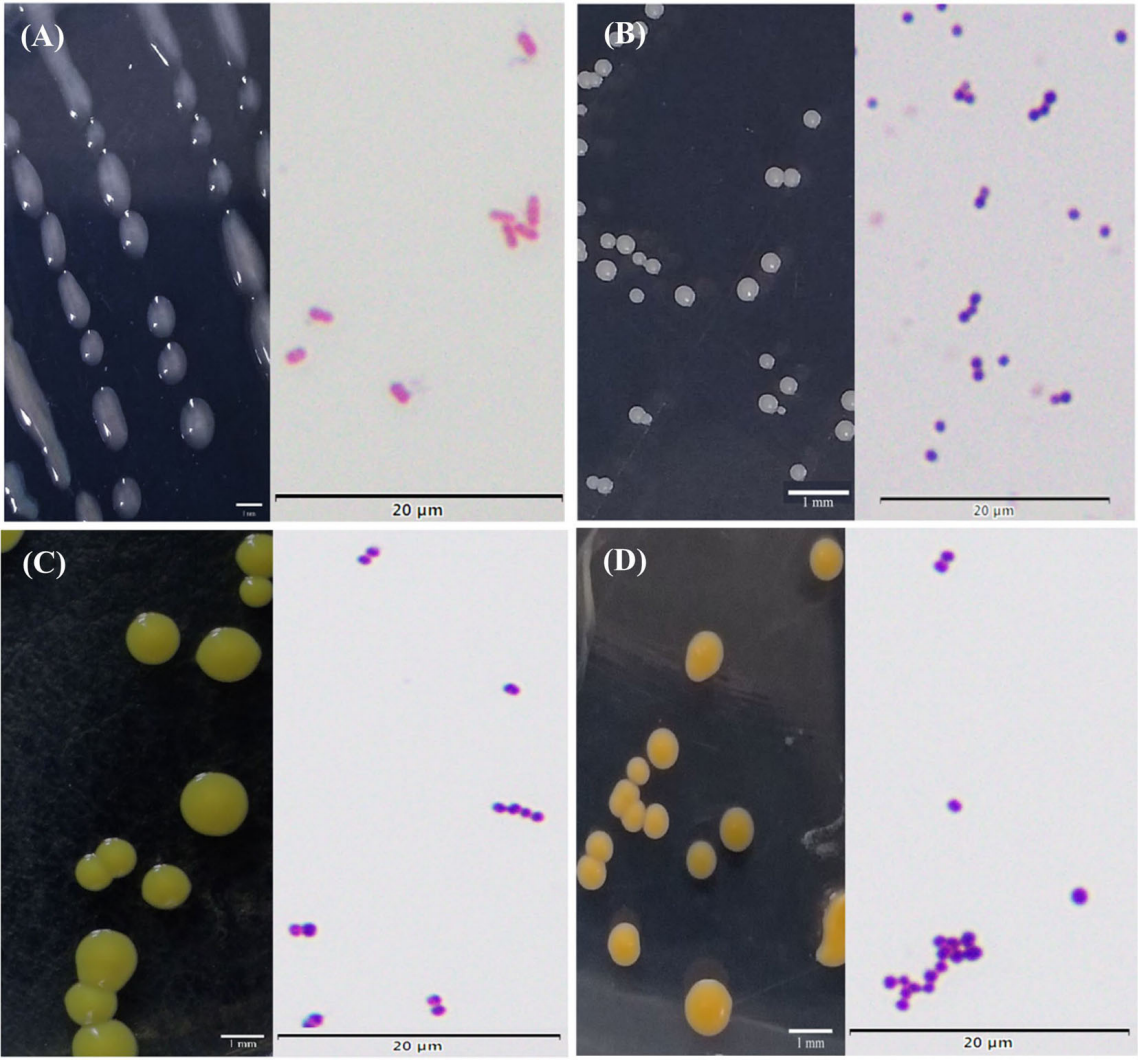
Bacterial colony morphology and Gram staining of the identified bacteria after purification: (A) R2A02–06, (B) R2A01–07, (C) LB01‐01 and (D) R2A02–09. Selected results are presented here, with the full dataset provided in the Supporting information.

**Figure 5:**
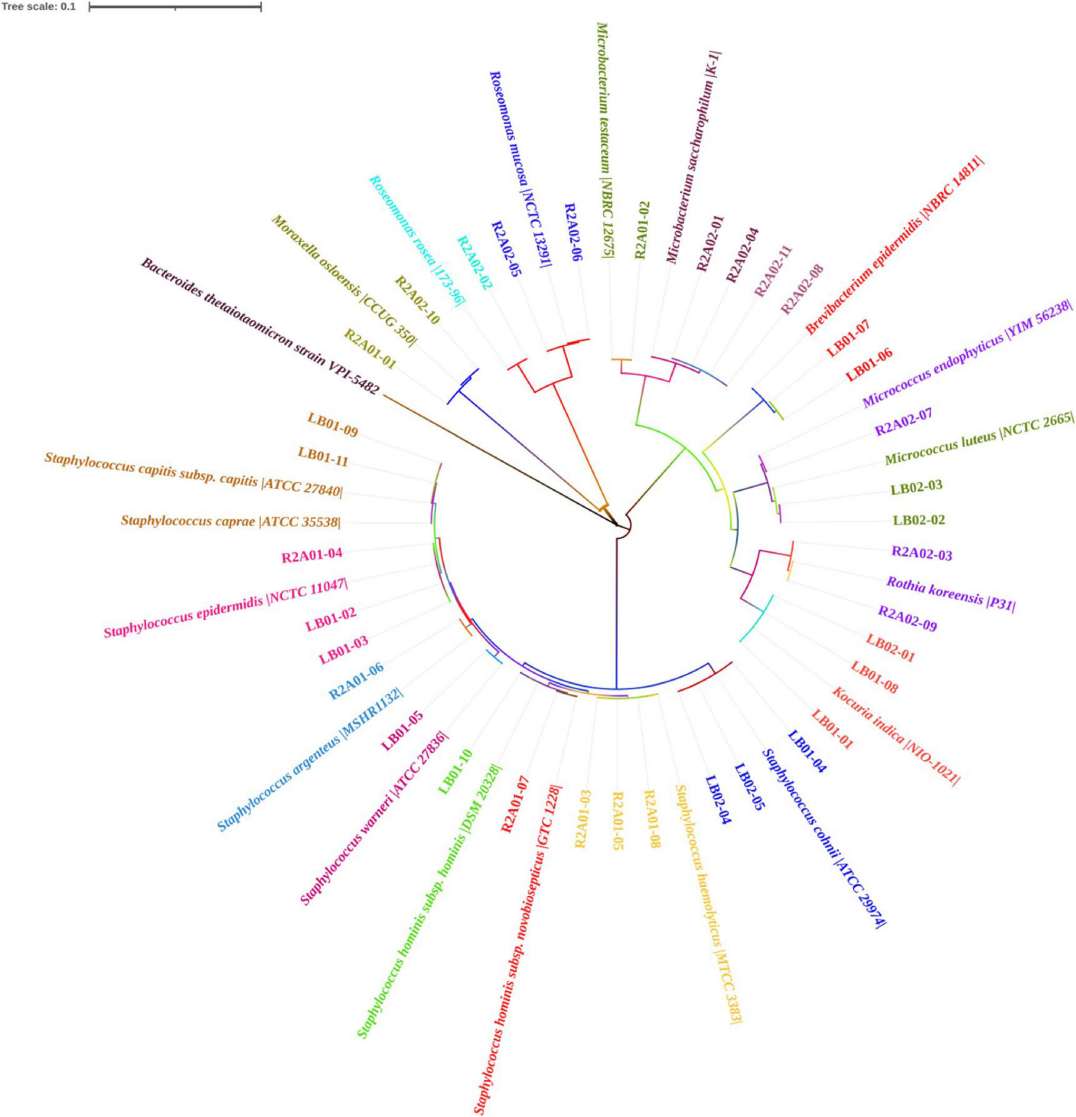
Neighbor‐joining phylogenetic tree of bacteria 16S rRNA sequence. The phylogenetic tree was calculated using Mega 7.0 software with *Bacteroides thetaiotaomicron* strain VPI‐5482 as the outgroup. It was constructed by the NJ method with the confidence values of each node based on 1000 bootstrap tests.

Volatile substances are produced by resident bacteria on the skin, which convert metabolites secreted in sweat into odorants, giving the human body a unique scent.^24^ Bacteria from the skin surface of the 8 volunteers were collected, cultured and strains purified, leading to preliminary morphological identification (Supporting information Table S1). The identified bacteria were selected for Gram Staining (Figs 4 and S1) and other subsequent experiments. Then, 35 bacteria were further characterized by PCR with primers 27F/1492R (Table S2) and sequencing. The products with a single band and a length of ≈1500 bp were sequenced (Fig. S2). The sequencing results of the 16S rRNA gene were treated with seqman. The sequence alignment results indicated that their similarity was >98% in ezbiocloud software (Table S3). The phylogenetic tree showed that the same species were clustered into one branch, which is consistent with the comparison results (Fig. 5).

### 3.6 Detection of volatile substances from bacteria derived from human skin

According to the results above, 3‐methyl‐1‐butanol, geranylacetone and nonanal are volatiles with an attractive effect on *F. (L.) taiwana* midges, and 6‐methyl‐5‐hepten‐2‐one was the most abundant volatile compound in human skin. To further detect which identified bacteria from human skin release these volatile substances, volatile substances from the cultured bacteria were collected and detected by GC–MS. The experiment showed that 18 types of bacteria produced a total of 37 major volatile compounds (Table S4; Fig. S3).

**Figure 6:**
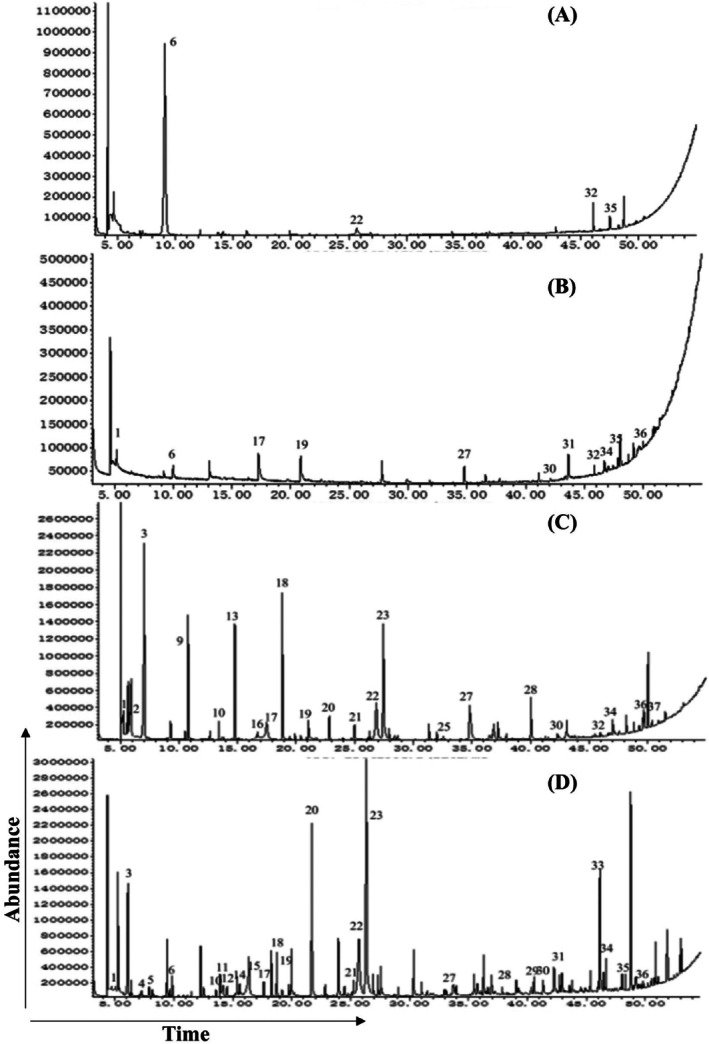
Total ion flow diagram of bacterial volatiles. The volatile substances from bacteria were detected by GC–MS. Different volatile substances released by 18 types of bacteria were found. The figure only displays partial results: (A) R2A01‐07, (B) LB02‐05, (C) LB02‐02 and (D) R2A02‐07. The vertical axis represents the abundance of the substance, and the horizontal axis represents the detection time.

3‐methyl‐1‐butanol was mainly produced by *Staphylococcus hominis* subsp. *Novobiosepticus* (R2A01‐07) with a high rate of 81.898% [Fig. 6(A)]. The proportion of produced 3‐methyl‐1‐butanol from *Microbacterium saccharophilum* (R2A02‐01) and *Staphylococcus cohnii* (LB01‐04) was also relatively high [Fig. 6(B)]. Geranylacetone was released by two types of bacteria (Table S4)–*Micrococcus luteus* (LB02‐02) and *Staphylococcus haemolyticus* (R2A01–08)–but the relative content of the volatile was relatively small [Fig. 6(C)]. Nonanal could be produced by four types of bacteria, *Micrococcus endophyticus* (R2A02‐07), *Staphylococcus hominis* subsp. *hominis* (LB01‐10), *Micrococcus luteus* (LB02‐02) and *Roseomonas mucosa* (R2A02‐06) [Fig. 6(D)]. 6‐methyl‐5‐hepten‐2‐one could be released by various bacteria such as *Micrococcus endophyticus* (R2A02‐07) and *Micrococcus luteus* (LB02‐02) [Fig. 6(C), (D)]. It was found that *Micrococcus luteus* (LB02‐02) and *Micrococcus endophyticus* (R2A02‐07) produce a greater variety of volatile compounds [Fig. 6(D); Table S4]. 3‐methyl‐1‐butanol was produced by five types of bacteria, 6‐methyl‐5‐hepten‐2‐one was produced by 12, nonanal by four and geranylacetone by six types of bacteria.

## 4 DISCUSSION

Midges are medically significant insects that pose significant public health concerns through their blood‐feeding behavior. The blood‐sucking preference of insects is mainly related to the type and content of host odor substances.^15^, ^17^ Insect antennae are one of the most sensitive and selective chemical sensing organs. They sense the presence of specific volatile compounds through receptors.^33^ Blood‐sucking insects can successfully locate their hosts and achieve blood‐sucking with antennae containing olfactory neuron receptors.^34^, ^35^ Currently, EAG was used to detect the stimulus response of insects to volatile compounds from numerous studies. *Crioceris duodecimpunctata* (*L*.) exhibited the highest EAG value to (*Z*)‐3‐hexen‐1‐ol.^36^ Locust antennae showed a strong stimulation response to volatile compounds, such as trans‐2‐Hexen‐1‐al, cis‐3‐Hexenyl acetate and decanal.^37^ 2,4‐Di‐tert‐butylphenol could interacted with the W^146^ residue of the Orco subunit in the antennae of *Drosophila melanogaster* with a concentration‐dependent and voltage‐ independent activation manner.^38^ It was found with EAG detection that the antennae of *F. (L.) taiwana* midges significantly respond to the human volatile substances including geranylacetone, 6‐methyl‐5‐hepten‐2‐one, decyl aldehyde and nonanal. The stimulus response increased with the increase of concentration of the volatile substances. By contrast, 3‐methyl‐1‐butanol and heptaldehyde had a smaller stimulation on the antennae of the midge. The results showed that the antennae of the *F. (L.) taiwana* midge play an important role in recognizing host volatiles in different concentration ranges. It is necessary to further determine the structure and functional characteristics of the internal sensors of antennae for more precise localization of odor receptors.

Insects mainly rely on the scent emitted by their hosts to search for them. The differences of mosquito attraction to humans are mainly the result of different volatile substances emitted by the human body. The different volatiles could influence olfactory signals of insects.^39^ It was found that the types of volatile compounds present on human skin are similar, but their relative content varies by individual. The results of our work are similar to those in a previous study.^40^ It was found that (*E*)‐2‐hexenal, (*E*)‐2‐heptenal, (*E*)‐2‐octenal and (*E*)‐2‐nonenal can induce repellent responses of *Culicoides nubeculosus*.^32^ Children infected with *Plasmodium* became more attractive to *Anopheles gambiae*, which is caused by a significant increase in the levels of aldehydes heptanal, octanal and nonanal in their skin volatiles.^41^ Host‐seeking *C. nubeculosus* were attracted by 6‐methyl‐5‐hepten‐2‐one at a concentration of 10^−10^ g per 10 µL on a filter paper (1 x 1 cm), whereas the biting midges were driven away by 6‐methyl‐5‐hepten‐2‐one at a concentration of 10^−8^ g per 10 µL on a filter paper. The behavioral response of insects to volatiles mainly depends on the concentration of volatile compounds.^9^ It was found that a mixture of 6‐methyl‐5‐hepten‐2‐one and geranylacetone showed significant repellency in the field.^42^ The discrepancies in the results may be attributed to the following factors: (I) differences in the concentrations and mixtures of compounds employed; (II) species‐specific responses to the different compounds; and (III) their experiments were conducted under field conditions, whereas our ethological experiments were performed in the laboratory. It is believed that the concentration of volatiles determines the behavioral response of insects, which is consistent with the results of this study. 0.001% 3‐methyl‐1‐butanol, 0.001%, 0.0001% and 0.00001% decyl aldehyde, 0.01% and 0.001% geranylacetone, and 0.00001% nonanal showed strong attractive effects on *F. (L.) taiwana* midges, whereas higher and lower concentrations showed few attractive effects or even repellent effects. Therefore, the results indicate that the behavioral response depends on the concentration of the tested volatile compounds, and midges prefer hosts with appropriate concentrations of volatile compounds.

Human odors produced by the skin microbiota can attract insects and microbiota in gut could induce changes in insect olfactory system.^28^, ^43^ Various volatile compounds were produced from human sweat by microorganisms. The released levels of geranylacetone, nonanal, decyl aldehyde and 6‐methyl‐5‐hepten‐2‐one are relatively higher in the volatile compounds produced by human skin microbiota.^44^ It was found abundant acidic volatile compounds were produced from human sweat decomposed by skin microbiota. These acidic volatile compounds play an important role in the host localization and blood‐sucking behavior of *Ae. aegypti* mosquitoes. Individuals who release more lactic acid in sweat have a stronger attraction to mosquitoes.^45^

It was found that binary mixtures of l‐lactic acid and chloroalkanes consistently synergized attraction in female *Ae. aegypti* mosquitoes (similar to l‐lactic acid + CO₂), whereas mixtures with sulfides showed less synergism.^46^

Additionally, the *Bacillus* in the skin microbiota of virus‐infected mice significantly increased. *Bacillus* has a strong ability to produce acetophenone, serving as a mosquito attractant, which ultimately regulates the attraction of virus‐infected mice to *Ae. aegypti* mosquitoes.^28^ Heptaldehyde, octanal, nonanal and decyl aldehyde released by *Beauveria bassiana* and *Metarhizium acridum* can attract aphids.^47^ Metabolites in human skin could be decomposed into butyl acetate, 2‐methylbutanoate, butyl butyrate, 3‐methyl‐1‐butanol and 3‐methylbutyric acid by *Staphylococcus epidermidis*, which are attractive to mosquitoes.^48^ In this study, 3‐methyl‐1‐butanol, which is suitable for use as an attractant for *F. (L.) taiwana* midges, was mainly produced by *Staphylococcus hominis* subsp. *novobiosepticus*, accounting for 81.898%. Geranylacetone and 6‐methyl‐5‐hepten‐2‐one could be produced by various bacteria such as *Micrococcus luteus* and *Staphylococcus haemolyticus*, nonanal could be produced by *Micrococcus endophyticus*. However, the relative content of the last three volatile compounds is relatively lower. The results indicate that bacteria on the human skin can produce volatile substances, which could affect the host selection preference of *F. (L.) taiwana*. By identifying attractive volatile compounds emitted by the skin microbiota, we can develop drugs targeting these specific microbes. This targeted intervention allows for the modulation of an individual's insect‐attracting volatile profile, thereby offering a more effective strategy for preventing and controlling vector‐borne diseases.

## 5 CONCLUSIONS

In summary, midge antennae are the main organs with chemoreceptors used to detect host odors. Differences in host odor are the main reason for the blood‐sucking preference of *F. (L.) taiwana* midges. Volatile substances can attract or repel midges in the appropriate concentration range. The differences in human volatile compounds are mainly regulated by the skin microbiota, which indirectly changes the olfactory behavior of midges by regulating human odor. 0.001% geranylacetone is best among the tested volatile substances to attract *F. (L.) taiwana* midges. This volatile compound could be produced by various bacteria. However, further research is needed to understand the specific mechanisms underlying the host preference of midges.

**ABBREVIATIONS**
EAG: electroantennogram/electroantennography
GC–MS: gas chromatography–mass spectrometry
NJ: neighbour‐joining method
SEM: scanning electron microscope

## CONFLICT OF INTEREST

The authors have no financial conflicts of interest.

## AUTHOR CONTRIBUTIONS

Experimental design: TL, YZ, and XH. Experiment execution: YZ, DG, and HC. Data analysis: TL, YZ, and DG. Manuscript writing: TL, YZ, and XH. All authors contributed to the article and approved the submitted version.

## ETHICS STATEMENT

All procedures involving the use of experimental animals were approved by the Animal Ethics Committee of Zunyi Medical University.

## Supporting information


**Figure S1.** Bacterial colony morphology and Gram staining. Scale bar, 20 μm.
**Fig. S2.** Results of PCR product amplification of bacterial 16S rRNA gene.
**Fig. S3.** Total ion flow diagram of bacterial volatiles.

**Table S1.** 16S rRNA gene amplification primer sequences.
**Table S2.** Preliminary identification results of bacterial morphology.
**Table S3.** 16S rRNA sequence alignment results in EzBiocloud database.
**Table S4.** Analysis results of volatile compounds of bacteria.

## Data Availability

Data sharing not applicable to this article as no datasets were generated or analysed during the current study.

## References

[1] Wang SC , Ching YH , Krishnaraj P , Chen GY , Radhakrishnan AS , Lee HM *et al*., Oogenesis of hematophagous midge *Forcipomyia taiwana* (Diptera: *Ceratopogonidae*) and Nuage localization of vasa in germline cells. Insects 11:106 (2020). 32033475 10.3390/insects11020106 PMC7074065

[2] He Y , Meng J , Li N , Li Z , Wang D , Kou M *et al*., Isolation of epizootic hemorrhagic disease virus serotype 10 from *Culicoides tainanus* and associated infections in livestock in Yunnan, China. Viruses 16:75 (2024). 10.3390/v16020175 PMC10892452 38399951

[3] Ching YH , Kuo YC , Su MC , Wang SC , Lin CF , Tu WC *et al*., Genetic differentiation of the bloodsucking midge *Forcipomyia taiwana* (Diptera: *Ceratopogonidae*): implication of the geographic isolation by the Central Mountain ranges in Taiwan. Insects 15:23 (2024). 38249029 10.3390/insects15010023 PMC10817045

[4] Wang HY , Takasaki T , Fu SH , Sun XH , Zhang HL , Wang ZX *et al*., Molecular epidemiological analysis of Japanese encephalitis virus in China. J Gen Virol 88:885–894 (2007). 17325361 10.1099/vir.0.82185-0

[5] Möhlmann TWR , Vogels CBF , Göertz GP , Pijlman GP , Ter Braak CJF , Te Beest DE *et al*., Impact of gut bacteria on the infection and transmission of pathogenic arboviruses by biting midges and mosquitoes. Microb Ecol 80:703–717 (2020). 32462391 10.1007/s00248-020-01517-6 PMC7476999

[6] Rebêlo JM , Rodrigues BL , Bandeira MD , Moraes JL , Fonteles RS and Pereira SR , Detection of *Leishmania amazonensis* and *Leishmania braziliensis* in *Culicoides* (Diptera, *Ceratopogonidae*) in an endemic area of cutaneous leishmaniasis in the Brazilian Amazonia. J Vector Ecol 41:303–308 (2016). 27860021 10.1111/jvec.12227

[7] Bernotienė R , Bartkevičienė G and Bukauskaitė D , The flying activity of biting midges (Ceratopogonidae: *Culicoides*) in Verkiai Regional Park, southeastern Lithuania. Parasitol Res 120:2323–2332 (2021). 33893548 10.1007/s00436-021-07147-2

[8] Bao S , Li G , Lu X , Lu T and Hou X , Identification and characterization of the Cul t 1 as major allergen from biting midge *Culicoides tainanus* . Mol Immunol 178:32–40 (2025). 39824033 10.1016/j.molimm.2025.01.004

[9] Isberg E , Bray DP , Birgersson G , Hillbur Y and Ignell R , Identification of cattle‐derived volatiles that modulate the behavioral response of the biting midge *Culicoides nubeculosus* . J Chem Ecol 42:24–32 (2016). 26687092 10.1007/s10886-015-0663-x

[10] Lu T , Ji Y , Chang M , Zhang X , Wang Y and Zou Z , The accumulation of modular serine protease mediated by a novel circRNA sponging miRNA increases *Aedes aegypti* immunity to fungus. BMC Biol 22:7 (2024). 38233907 10.1186/s12915-024-01811-6 PMC10795361

[11] Ji Y , Lu T , Zou Z and Wang Y , *Aedes aegypti* CLIPB9 activates prophenoloxidase‐3 in the presence of CLIPA14 after fungal infection. Front Immunol 13:927322 (2022). 35967454 10.3389/fimmu.2022.927322 PMC9365933

[12] Logan JG and Birkett MA , Semiochemicals for biting fly control: their identification and exploitation. Pest Manag Sci 63:647–657 (2007). 17549674 10.1002/ps.1408

[13] Pickett JA , Birkett MA , Dewhirst SY , Logan JG , Omolo MO , Torto B *et al*., Chemical ecology of animal and human pathogen vectors in a changing global climate. J Chem Ecol 36:113–121 (2010). 20119869 10.1007/s10886-010-9739-9

[14] Harrup LE , Logan JG , Cook JI , Golding N , Birkett MA , Pickett JA *et al*., Collection of *Culicoides* (Diptera: *Ceratopogonidae*) using CO_2_ and enantiomers of 1‐octen‐3‐ol in the United Kingdom. J Med Entomol 49:112–121 (2012). 22308779 10.1603/me11145

[15] Omolo MO , Ndiege IO and Hassanali A , Semiochemical signatures associated with differential attraction of *Anopheles gambiae* to human feet. PLoS One 16:e0260149 (2021). 34860850 10.1371/journal.pone.0260149 PMC8641859

[16] Díaz‐Santiz E , Rojas JC , Casas‐Martínez M , Cruz‐López L and Malo EA , Rat volatiles as an attractant source for the Asian tiger mosquito, *Aedes albopictus* . Sci Rep 10:5170 (2020). 32198359 10.1038/s41598-020-61925-z PMC7083917

[17] Bakker JW , Loy DE , Takken W , Hahn BH and Verhulst NO , Attraction of mosquitoes to primate odours and implications for zoonotic plasmodium transmission. Med Vet Entomol 34:17–26 (2020). 31420992 10.1111/mve.12402 PMC7002228

[18] Chen Z , Liu F and Liu N , Human odour coding in the yellow fever mosquito, Aedes aegypti. Sci Rep 9:13336 (2019). 31527631 10.1038/s41598-019-49753-2 PMC6746732

[19] Zhao Z , Zung JL , Hinze A , Kriete AL , Iqbal A , Younger MA *et al*., Mosquito brains encode unique features of human odour to drive host seeking. Nature 605:706–712 (2022). 35508661 10.1038/s41586-022-04675-4 PMC9725754

[20] Dormont L , Mulatier M , Carrasco D and Cohuet A , Mosquito Attractants. J Chem Ecol 47:351–393 (2021). 33725235 10.1007/s10886-021-01261-2

[21] Weeks ENI , Logan JG , Birkett MA , Caulfield JC , Gezan SA , Welham SJ *et al*., Electrophysiologically and behaviourally active semiochemicals identified from bed bug refuge substrate. Sci Rep 10:4590 (2020). 32165700 10.1038/s41598-020-61368-6 PMC7067832

[22] Drabińska N , Flynn C , Ratcliffe N , Belluomo I , Myridakis A , Gould O *et al*., A literature survey of all volatiles from healthy human breath and bodily fluids: the human volatilome. J Breath Res 15:034001 (2021). 10.1088/1752-7163/abf1d0 33761469

[23] Dormont L , Bessière JM and Cohuet A , Human skin volatiles: a review. J Chem Ecol 39:569–578 (2013). 23615881 10.1007/s10886-013-0286-z

[24] Coutinho‐Abreu IV , Riffell JA and Akbari OS , Human attractive cues and mosquito host‐seeking behavior. Trends Parasitol 38:246–264 (2022). 34674963 10.1016/j.pt.2021.09.012 PMC10789295

[25] Penn DJ , Oberzaucher E , Grammer K , Fischer G , Soini HA , Wiesler D *et al*., Individual and gender fingerprints in human body odour. J R Soc Interface 4:331–340 (2007). 17251141 10.1098/rsif.2006.0182 PMC2359862

[26] Verhulst NO , Andriessen R , Groenhagen U , Bukovinszkiné Kiss G , Schulz S , Takken W *et al*., Differential attraction of malaria mosquitoes to volatile blends produced by human skin bacteria. PLoS One 5:e15829 (2010). 21209854 10.1371/journal.pone.0015829 PMC3012726

[27] Ruiz‐López MJ , Mosquito behavior and vertebrate microbiota interaction: implications for pathogen transmission. Front Microbiol 11:573371 (2020). 33362732 10.3389/fmicb.2020.573371 PMC7755997

[28] Zhang H , Zhu Y , Liu Z , Peng Y , Peng W , Tong L *et al*., A volatile from the skin microbiota of flavivirus‐infected hosts promotes mosquito attractiveness. Cell 185:2510–2522 (2022). 10.1016/j.cell.2022.05.016 35777355

[29] Lucas‐Barbosa D , DeGennaro M , Mathis A and Verhulst NO , Skin bacterial volatiles: propelling the future of vector control. Trends Parasitol 38:15–22 (2022). 34548253 10.1016/j.pt.2021.08.010

[30] Showering A , Martinez J , Benavente ED , Gezan SA , Jones RT , Oke C *et al*., Skin microbiome alters attractiveness to *anopheles* mosquitoes. BMC Microbiol 22:98 (2022). 35410125 10.1186/s12866-022-02502-4 PMC9004177

[31] De Obaldia ME , Morita T , Dedmon LC , Boehmler DJ , Jiang CS , Zeledon EV *et al*., Differential mosquito attraction to humans is associated with skin‐derived carboxylic acid levels. Cell 185:4099–4116.e4013 (2022). 36261039 10.1016/j.cell.2022.09.034 PMC10069481

[32] Isberg E and Ignell R , Cattle‐derived unsaturated aldehydes repel biting midges and mosquitoes. J Chem Ecol 48:359–369 (2022). 35107692 10.1007/s10886-021-01347-x PMC9079034

[33] Elgar MA , Zhang D , Wang Q , Wittwer B , Thi Pham H , Johnson TL *et al*., Insect antennal morphology: the evolution of diverse solutions to odorant perception. Yale J Biol Med 91:457–469 (2018). 30588211 PMC6302626

[34] Ortega‐Insaurralde I and Barrozo RB , The closer the better: sensory tools and host‐association in blood‐sucking insects. J Insect Physiol 136:104346 (2022). 34896372 10.1016/j.jinsphys.2021.104346

[35] Pusawang K , Sriwichai P , Aupalee K , Yasanga T , Phuackchantuck R , Zhong D *et al*., Antennal morphology and sensilla ultrastructure of the malaria vectors, *Anopheles maculatus* and *an. Sawadwongporni* (Diptera: *Culicidae*). Arthropod Struct Dev 76:101296 (2023). 37657362 10.1016/j.asd.2023.101296 PMC10530502

[36] Pistillo OM , D'Isita I and Germinara GS , Olfactory response of the spotted asparagus beetle, *Crioceris duodecimpunctata (L.)* to host plant volatiles. J Chem Ecol 48:41–50 (2022). 34738203 10.1007/s10886-021-01323-5

[37] Wu H , Liu J , Liu Y , Abbas M , Zhang Y , Kong W *et al*., CYP6FD5, an antenna‐specific P450 gene, is potentially involved in the host plant recognition in *Locusta migratoria* . Pestic Biochem Physiol 188:105255 (2022). 36464360 10.1016/j.pestbp.2022.105255

[38] Lee S , Eom S , Pyeon M , Moon M , Yun J , Lee J *et al*., Identification of 2,4‐Di‐tert‐butylphenol as a novel agonist for insect odorant receptors. Int J Mol Sci 25:220 (2023). 38203390 10.3390/ijms25010220 PMC10779170

[39] Mweresa CK , Mukabana WR , Omusula P , Otieno B , Van Loon JJ and Takken W , Enhancing attraction of African malaria vectors to a synthetic odor blend. J Chem Ecol 42:508–516 (2016). 27349651 10.1007/s10886-016-0711-1

[40] Bernier UR , Kline DL , Barnard DR , Schreck CE and Yost RA , Analysis of human skin emanations by gas chromatography/mass spectrometry. 2. Identification of volatile compounds that are candidate attractants for the yellow fever mosquito (*Aedes aegypti*). Anal Chem 72:747–756 (2000). 10701259 10.1021/ac990963k

[41] Robinson A , Busula AO , Voets MA , Beshir KB , Caulfield JC , Powers SJ *et al*., Plasmodium‐associated changes in human odor attract mosquitoes. Proc Natl Acad Sci U S A 115:E4209–e4218 (2018). 29666273 10.1073/pnas.1721610115 PMC5939094

[42] Logan JG , Seal NJ , Cook JI , Stanczyk NM , Birkett MA , Clark SJ *et al*., Identification of human‐derived volatile chemicals that interfere with attraction of the Scottish biting midge and their potential use as repellents. J Med Entomol 46:208–219 (2009). 19351071 10.1603/033.046.0205

[43] Li H , Yu Y , Zhang J , Wang Y , Zhang L , Zhai J *et al*., Gut microbiota influences feeding behavior via changes in olfactory receptor gene expression in Colorado potato beetles. Front Microbiol 14:1197700 (2023). 37455752 10.3389/fmicb.2023.1197700 PMC10338844

[44] Michalet S , Minard G , Chevalier W , Meiffren G , Saucereau Y , Tran Van V *et al*., Identification of human skin bacteria attractive to the Asian Tiger mosquito. Environ Microbiol 21:4662–4674 (2019). 31464044 10.1111/1462-2920.14793

[45] Raji JI , Melo N , Castillo JS , Gonzalez S , Saldana V , Stensmyr MC *et al*., *Aedes aegypti* mosquitoes detect acidic volatiles found in human odor using the IR8a pathway. Curr Biol 29:1253–1262.e1257 (2019). 30930038 10.1016/j.cub.2019.02.045 PMC6482070

[46] Bernier UR , Kline DL , Allan SA and Barnard DR , Laboratory studies of *Aedes aegypti* attraction to ketones, sulfides, and primary Chloroalkanes tested alone and in combination with L‐lactic acid. J Am Mosq Control Assoc 31:63–70 (2015). 25843177 10.2987/14-6452R.1

[47] Fingu‐Mabola JC , Martin C , Bawin T , Verheggen FJ and Francis F , Does the infectious status of aphids influence their preference towards healthy, virus‐infected and Endophytically colonized plants? Insects 11:435 (2020). 32664588 10.3390/insects11070435 PMC7412421

[48] Verhulst NO , Mbadi PA , Kiss GB , Mukabana WR , van Loon JJ , Takken W *et al*., Improvement of a synthetic lure for *Anopheles gambiae* using compounds produced by human skin microbiota. Malar J 10:28 (2011). 21303496 10.1186/1475-2875-10-28 PMC3041721

