# Supplementary material for: The effects of human volatiles produced by skin microbiota on Forcipomyia (Lasiohelea) taiwana host preference
Source: Pest Manag Sci. 2025 Jul 30;81(12):7815–26. doi: 10.1002/ps.70089 (PMC12618916; doi:10.1002/ps.70089)
Supplement: Supplementary file 1 — Figure S1. Bacterial colony morphology and Gram staining. Scale bar, 20 μm. Fig. S2. Results of PCR product amplification of bacterial 16S rRNA gene. Fig. S3. Total ion flow diagram of bacterial volatiles. [file PS-81-7815-s001.docx]

## Supplementary figures


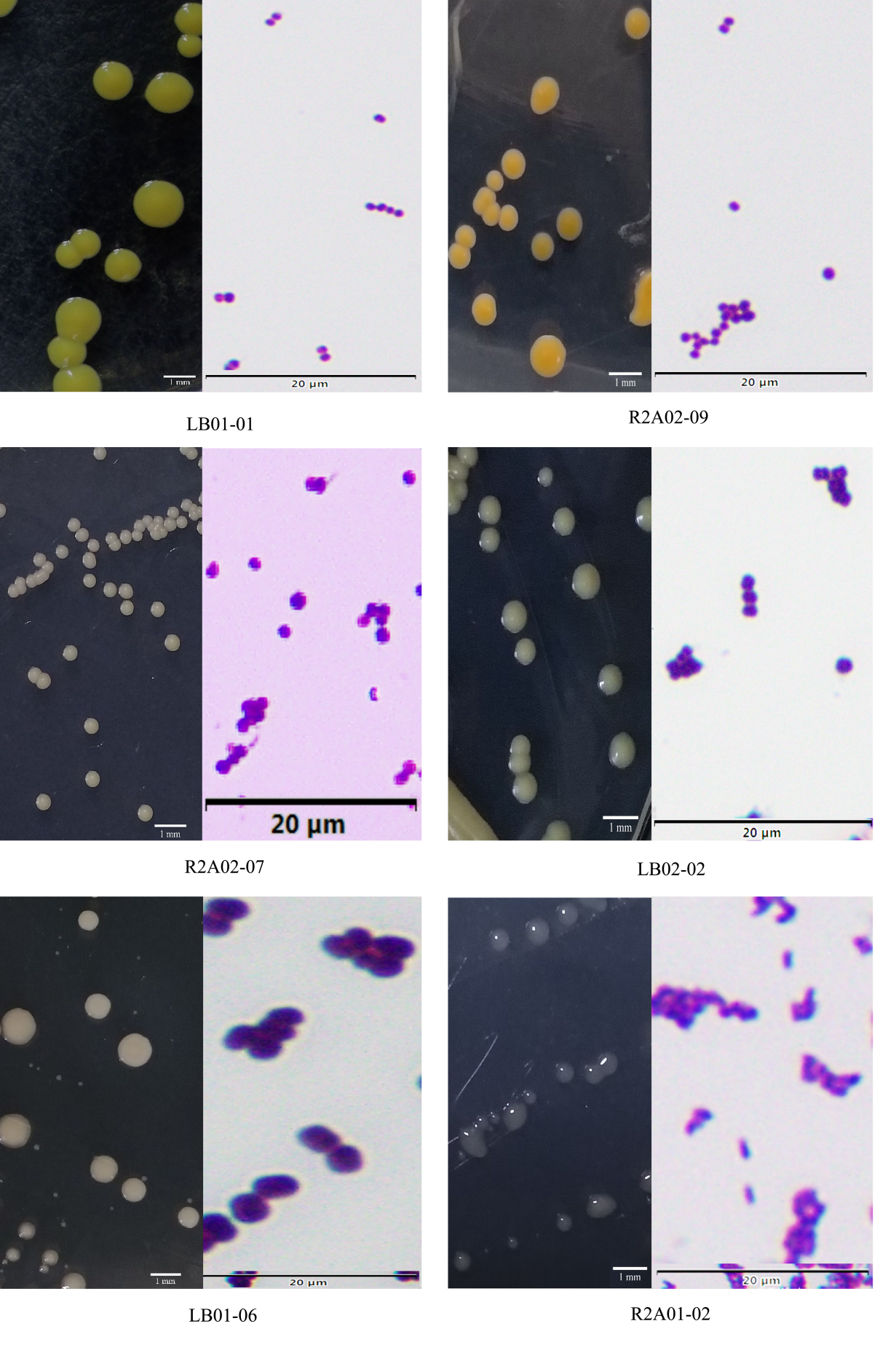

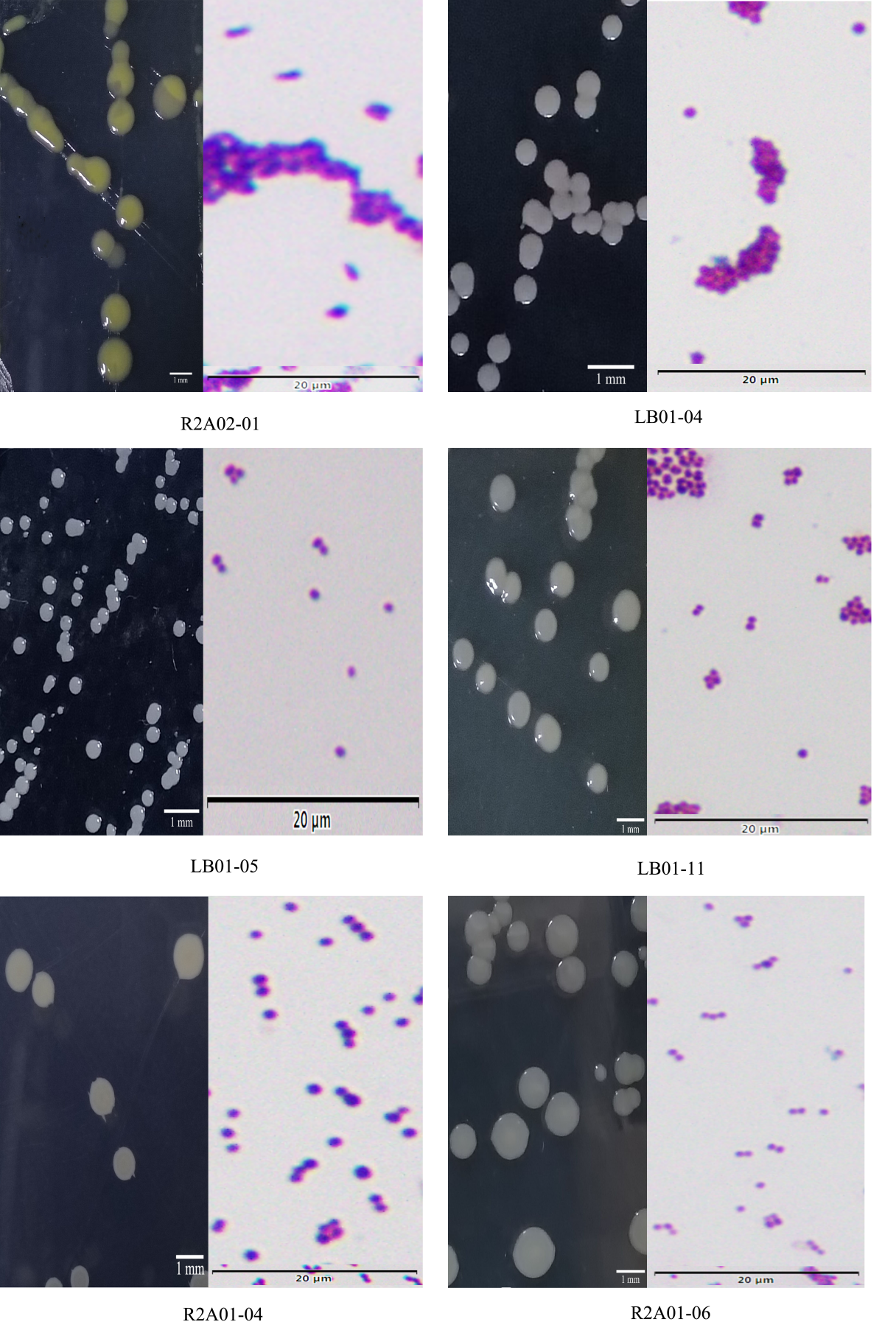

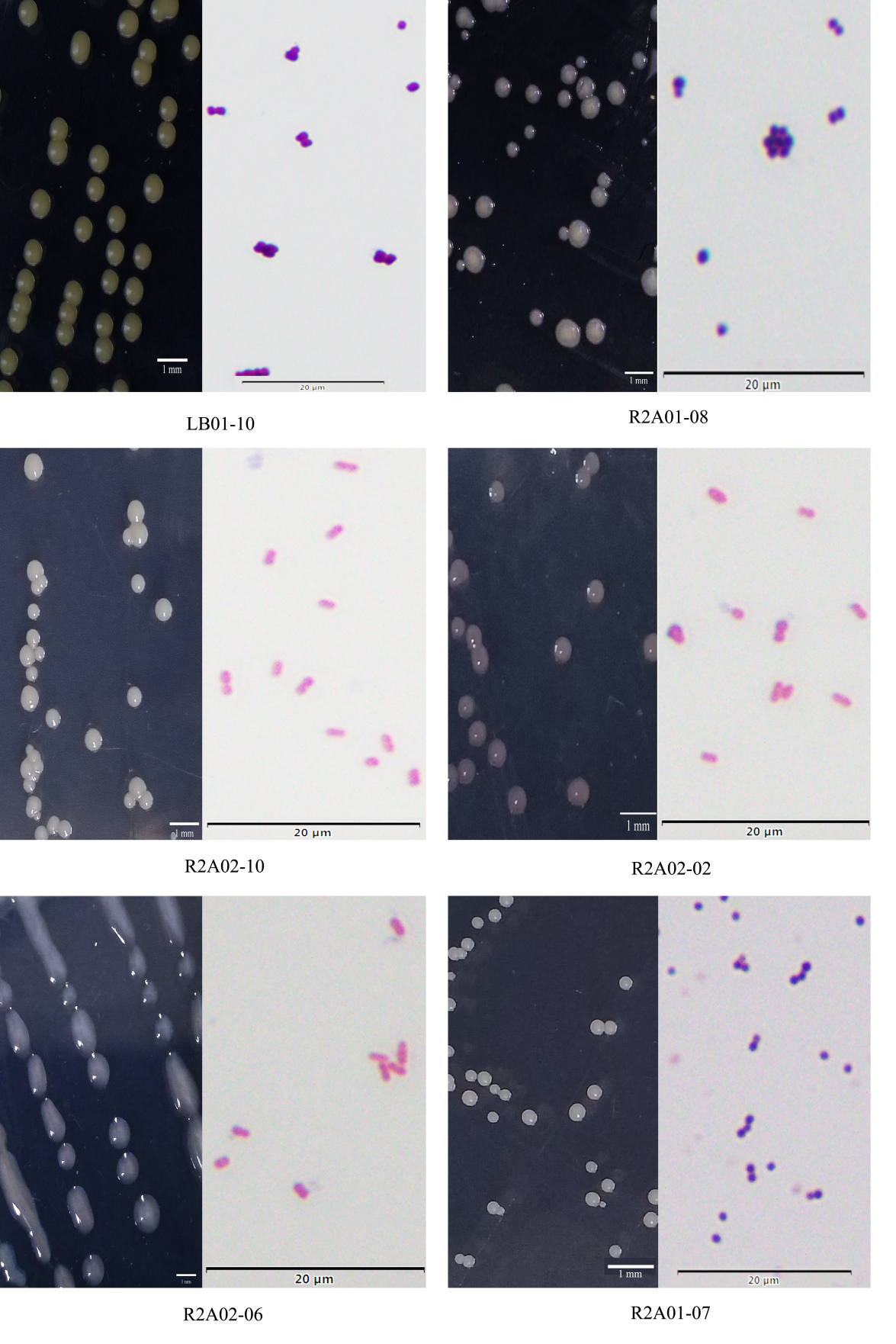


**Figure S1. Bacterial colony morphology and Gram staining**

The purified bacterial strains after 6 rounds of purification were conducted preliminary morphological identification. The identified bacteria were selected for Gram Staining. Scale bar, 20 μm.


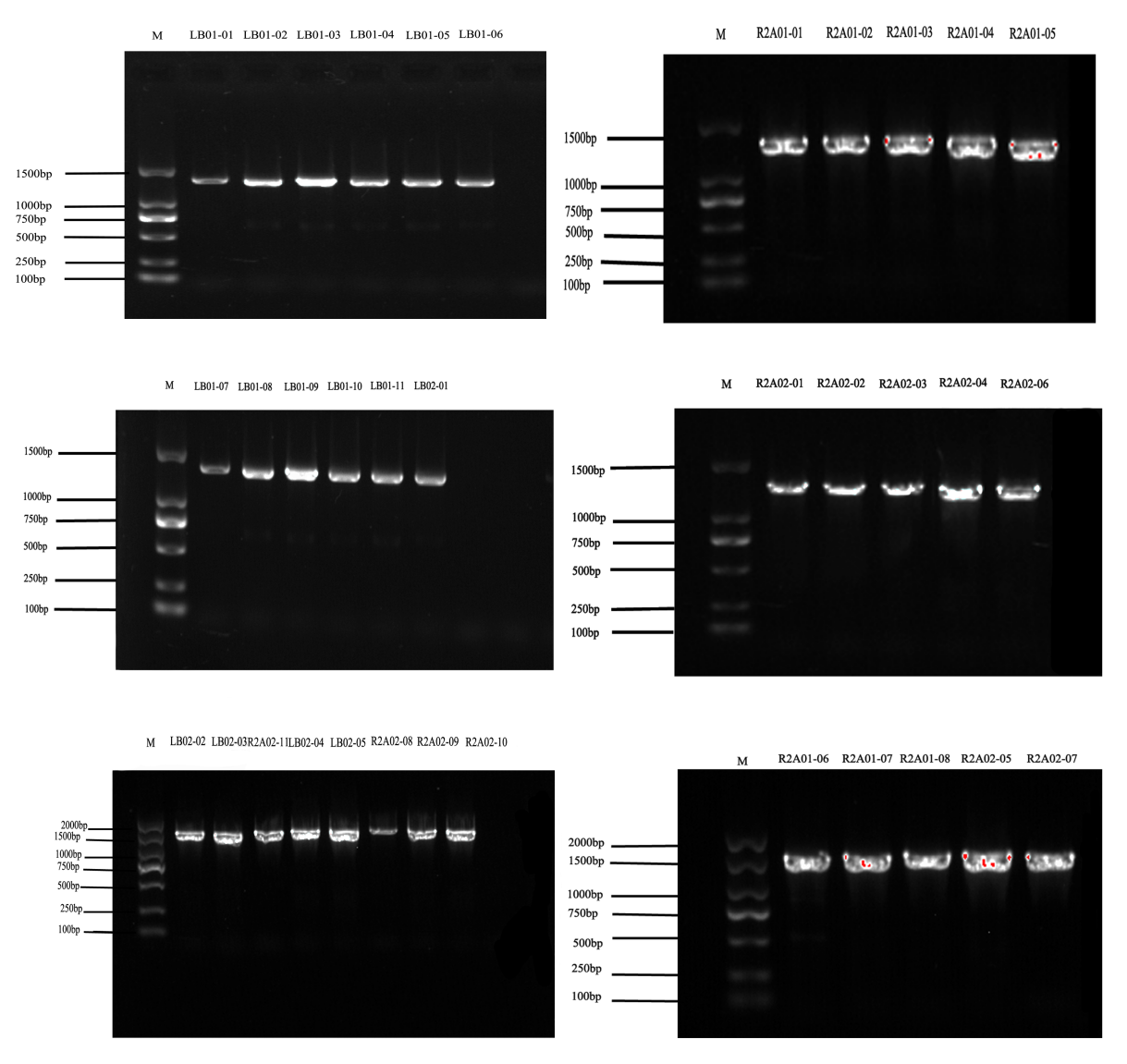


**Figure S2. Results of PCR product amplification of bacterial 16S rRNA gene**


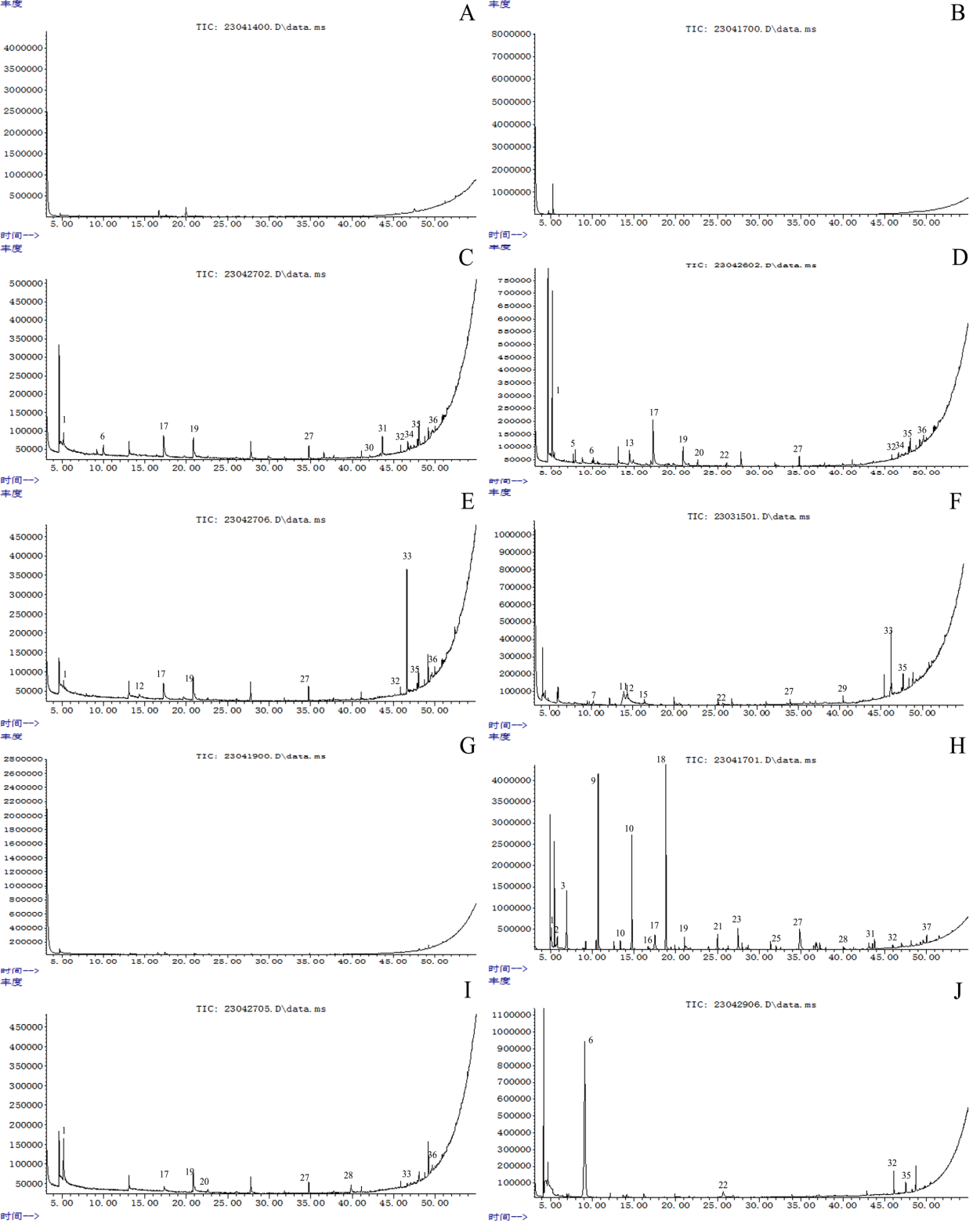


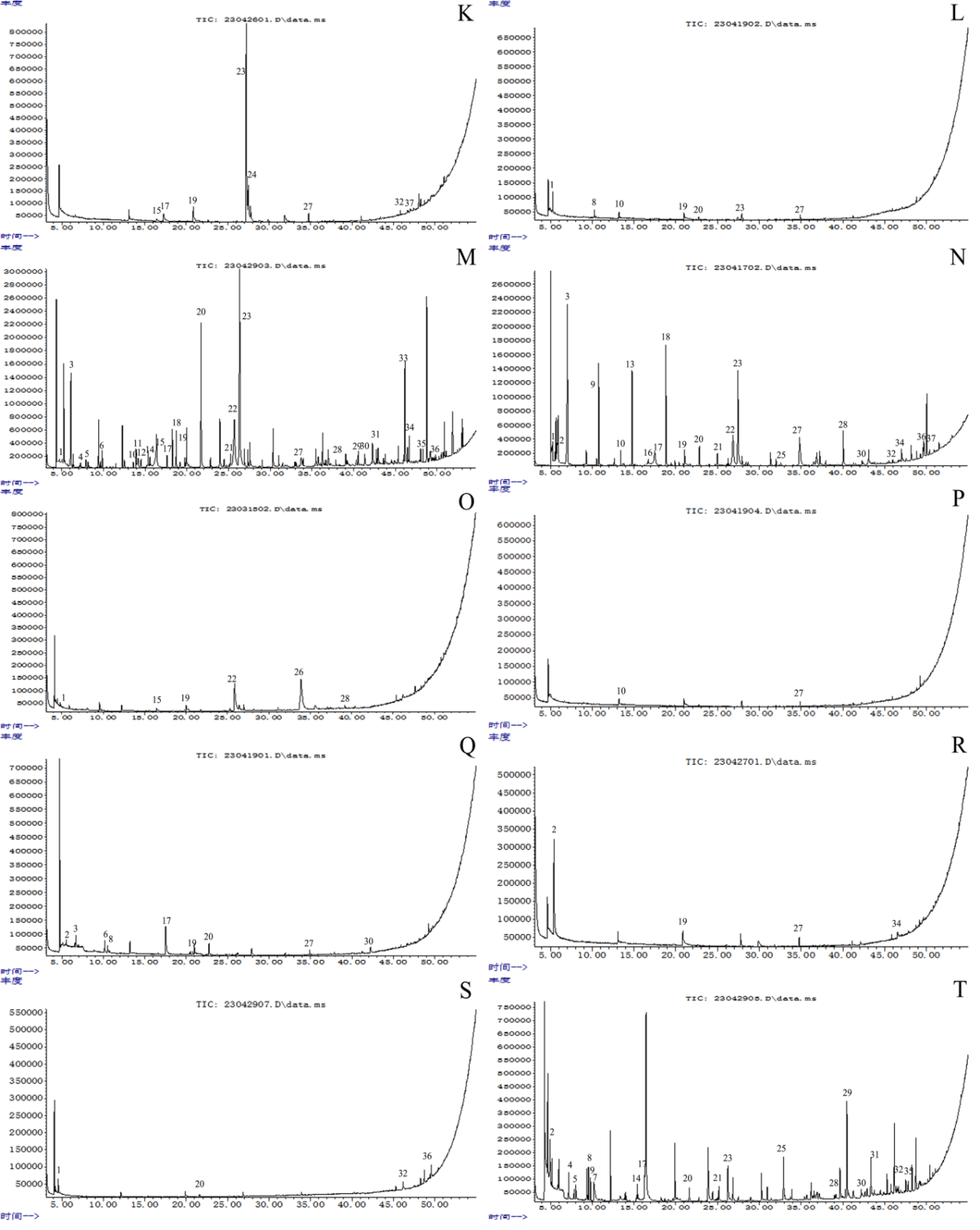


**Figure S3. Total ion flow diagram of bacterial volatiles**

(A) LB blank. (B) R2A blank. (C) LB01-04. (D) LB01-05. (E) LB01-11. (F) R2A01-04. (G) R2A01-06. (H) LB01-10. (I) R2A01-08. (J) R2A01-07. (K) LB01-01. (L) R2A02-09. (M) R2A02-07. (N) LB02-02. (O) LB01-06. (P) R2A01-02. (Q) R2A02-01. (R) R2A02-10. (S) R2A02-02. (T) R2A02-06.
